# The effect of *Alpinia oxyphylla* essential oil on growth performance, immune, antioxidant functions and gut microbiota in pigs

**DOI:** 10.3389/fvets.2024.1468520

**Published:** 2024-12-10

**Authors:** Fengming Chen, Zhimou Liu, Chun Xie, Jieyi He, Jiayi Chen, Kaiqiang Peng, Xu Chen, Jiajia He, Zhenyi Liu, Hui Yang, Kelang Kang, Binsheng He, Qian Lin

**Affiliations:** ^1^Hunan Provincial Key Laboratory of the Traditional Chinese Medicine Agricultural Biogenomics, Changsha Medical University, Changsha, Hunan, China; ^2^Institute of Bast Fiber Crops, Chinese Academy of Agricultural Sciences, Changsha, China; ^3^Hunan Nuoz Biological Technology Co., Ltd., Yiyang, Hunan, China; ^4^College of Animal Science and Technology, Hunan Agricultural University, Changsha, China; ^5^Hunan Academy of Agricultural Sciences, Changsha, China

**Keywords:** *Alpinia oxyphylla*, essential oil, antioxidation capacities, immune function, gut microbiota, fattening pig

## Abstract

*Alpinia oxyphylla*, a perennial herb belonging to the Zingiberaceae family, has a long history of traditional medicinal use. The present study evaluated the efficacy of different concentrations of *Alpinia oxyphylla* essential oil (AEO) on the growth performance, serum antioxidation capacities, immune function, apparent digestibility of nutrients, and gut microbiota in fattening pigs. A total of 120 pigs were divided into five treatments, with six replicates each and four pigs per replicate. The pigs were fed a basal diet or basal diet with chlortetracycline (CTC) alone or AEO at 250, 500, and 1,000 mg/kg (referred to as groups AEO1, AEO2, and AEO3, respectively) for 35 days, preceded by a 7-day pre-feed period. The results show that there were no statistically significant differences in growth performance for any dose of AEO supplementation. AEO increased L-DLC content, total protein content and the activity of GSH in serum (*p* < 0.05). The AEO also exhibited a linear increase in serum IgG content (*p* < 0.05). Dietary supplementation with AEO improved apparent digestibility of crude ash and calcium (*p* < 0.05). In gut microbiota, AEO modified the diversity and abundance of bacterial communities in fattening pigs. The abundance of *Dorea*, *Blautia*, *Butyricicoccus*, *Bulleidia*, and *Lactobacillus* was higher in the AEO groups compared to the control group, while *Clostridium* and *Turicibacter* were lower. The *Bifidobacteriales* and *Pseudomonas* were abundant in group AEO1 and AEO3, respectively. In conclusion, dietary supplementation of 1,000 mg/kg AEO has the potential to improve growth performance, immunological, biochemical, and antioxidant statuses. Additionally, AEO can increase the efficiency of nutrient digestion and absorption through the regulation of gut microbiota.

## Introduction

Over the past few decades, antibiotics have been widely used in livestock as antibiotic growth promoters (AGPs). Small quantities of antibiotics in animal feed were sufficient to promote growth and prevent diseases. However, the AGPs enter soil and aquatic ecosystems, leading to antibiotic pollution and the emergence of antibiotic resistance eventually (1, 2). After the European Union and the United States implemented a ban on AGPs, China also followed suit in 2020. Currently, the ban on AGPs continues to motivate the livestock industry to explore appropriate antibiotic alternatives that can support animal development and welfare (3), and growth-promoting alternatives to antibiotics in livestock production have become a subject of intense research.

Essential oils (EOs) are mixtures of volatile and hydrophobic compounds extracted from plants (4). They have the ability to enhance animal immunity, ameliorate intestinal function and promote growth, making them a viable alternative to dietary antibiotics in animals. Regularly, multiple concurrent environmental stressors, such as weaning, bacterial, viral infection and heat, continually impose extensive pressures to pigs, and these stress results in oxidative stress, which adversely affects the growth performance and health of pigs (5, 6). EOs contain flavonoids, phenols, aldehydes, and their derivatives, all of which have strong antioxidant and anti-inflammatory activities (7). These properties help cells defend against inflammation and oxidative damage caused by reactive oxygen species (ROS), thereby maintaining homeostasis (8, 9). Moreover, EOs have been reported to maintain the stability of the intestinal environment and improve relative abundance of probiotics, such as *Lactobacillus* and *Bifidobacterium* (10, 11). These probiotics positively impact gut health and ultimately contribute to improved growth performance.

*Alpinia oxyphylla*, a perennial herb belonging to the *Zingiberaceae* family, is native to the tropical and subtropical regions of China (12). It has a long history of food and traditional medicinal use in China spanning centuries (13), and is widely cultivated in southern China (14). Essential oil extracted from *Alpinia oxyphylla* is abundant in flavonoids, diarylheptanoids, terpenoids, volatile oils, steroids, and other glycosides (15, 16). These contents in AEO were further confirmed to have anti-hyperuricemic, and other therapeutic mechanisms for disease. In addition, increasing evidence indicates AEO has diverse bioactivities, including antioxidant (17), antitumor (14), anti-inflammatory (18), modulating the gut microbiota (19), protecting intestinal barrier integrity (20), and exhibiting bacteriostasis (21). These characteristics make AEO both cost-effective and easily available, highlighting its potential value as a feed additive. However, few studies have evaluated the effects of AEO on fattening pigs, which limits the use of *Alpinia oxyphylla* in the animal industry as an alternative to antibiotic growth promoters. In addition, there is a scarcity of data on the effects of AEO on the gut microbiota of pigs. Consequently, the aim of the study is to evaluate the efficacy of different concentrations of AEO in the growth performance, serum antioxidation capacities, immune function, apparent nutrient digestibility, and gut microbiota in fattening pigs, in order to provide data supporting the rational use of AEO.

## Materials and methods

### Ethical approval

All experimental procedures were reviewed and approved by the animal care committee of Changsha Medical University (No. 2023-01), Changsha, China.

### Animals, diets, and treatments

The study employed a single-factor randomized trial design. A sample of 120 male fattening pigs (Landrace × Yorkshire crossbreed pigs (M1) crossed with Duroc (DJ) breed boars), all belonging to the same breed and having similar health and body weight, were randomly assigned to five treatment groups (with six replicates per treatment and four pigs per replicate). Furthermore, pigs were subjected to a pre-fed regimen for 7 days, then the formal test spanned 35 days. The distribution of groups is shown in Table 1.

**Table 1:** Experimental design and grouping.

Group	Diet
Con	Basal diet
CTC	Basal diet +75 mg/kg chlortetracycline (15%) premix
AEO1	Basal diet +250 mg/kg AEO
AEO2	Basal diet +500 mg/kg AEO
AEO3	Basal diet +1,000 mg/kg AEO

The corn-soybean meal basal diet was designed based on the National Research Council (NRC, 2012), and the ingredients and nutritional levels are shown in Table 2. Pigs were provided with a pelleted feed throughout the entire period. The AEO was purchased from Hunan Nuoz Biological Technology Co., Ltd.

**Table 2:** Composition and nutrition levels of the basal diet (air-dry basis, %).

Item	Content
Ingredients	
Corn	27.50
Rice bran	10.00
Rice bran meal	16.00
Broken rice	30.00
Soybean meal	12.50
Premix^a^	4.00
Total	100.00
Nutrient level^b^	
DE (Mcal/kg)	3.20
CP	15.02
Ca	0.55
TP	0.76
AP	0.20
Lys	1.15
Thr	0.60
Met	0.25
Met Cys	0.50

a Premix contained per kg VA, 325 IU; VD, 37.5 IU; VE, 2.75 IU; VK3, 0.013 mg; VB2, 0.63 mg; VB6, 0.25 mg; VB12, 2.5 mg; biotin, 0.013 mg; folic acid, 0.08 mg; D-pantothenic acid, 2.00 mg; hydrochloric acid, 2.5 mg; choline chloride, 0.08 mg; antioxidants, 12.50 mg; FeSO4.H2O, 12.50 mg; CuSO4.H2O, 0.88 mg; ZnO, 15.00 mg; MnSO4.H2O, 0.50 mg; Na2SeO3 0.04 mg; Kl, 0.04 mg.

b Digestible energy was a calculated value.

### Sampling and collection

In the present study, the methods and parameters collected were according to our previous study (6). Pigs were weighed on an empty stomach at the beginning and the end of the experiment, and average daily gain (ADG) was calculated. Feed intake in each repeated was recorded to calculate average daily feed intake (ADFI) and feed-to-weight ratio (F/G). Moreover, 500 g of feed samples for each treatment were collected, and 1 kg of fresh and uncontaminated fecal samples for each replicate were randomly obtained using disposable sterile gloves. Samples were stored for testing.

### Serum biochemical parameters

The serum biochemical parameters were measured with an automatic biochemical analyzer (Hitachi automatic biochemical analyzer 7,000, Japan) including aspartate aminotransferase (AST), alanine aminotransferase (ALT), alkaline phosphatase (ALP), urea, glucose (GLU), total cholesterol (TC), triglyceride (TG), low-density lipoprotein (LDL), high-density lipoprotein (HDL), total protein (TP), albumin (ALB) and globulin (GLB) (22). Six antioxidant indicators were measured with a microplate reader FluoStar Optima (BMG Labtech, Offenburg, Germany), including superoxide dismutase (SOD), glutathione peroxidase (GPX), catalase (CAT), glutathione (GSH), total antioxidant capacity (T-AOC) and malondialdehyde (MDA) contents, in accordance with our previous method (23). The contents of IgA, IgG, and IgM and complements C3 and C4 in serum were determined by the enzyme-linked immunosorbent assay (ELISA, Nanjing Jiancheng Bioengineering Institute, Nanjing, China).

### Nutrient digestibility determination

Acid insoluble ash (AIA) was used as a digestibility indicator to determine the apparent total tract digestibility including dietary dry matter (DM), energy, crude protein (CP), crude fat (*CF*), crude fiber (*CF*), crude ash, calcium, and phosphorus.

### Microbial DNA extraction and PCR amplification

As previously described, the extraction of DNA from fecal specimens and the amplification of 16S ribosomal RNA were conducted in accordance with Wang et al. (24). DNA was isolated from the fecal samples utilizing the E.Z.N.A.^®^ Soil DNA Kit (Omega Biotek, Norcross, GA, United States) following the manufacturer’s protocol. The V3–V4 region of 16S rRNA in bacterial populations was amplified employing universal primers 338F/806R, and the samples were subsequently sequenced on the Illumina Miseq PE300 platform (Illumina, San Diego, CA, United States). Raw sequence data were filtered and denoised initially, and regions of low quality within the sequences were subsequently excised and excluded. The QIIME2 software (version 2019.4) facilitated the creation of a table of amplicon sequence variants (ASVs) and aided in the construction of rarefaction curves along with analyses of both alpha and beta diversity. For the taxonomic classification, the Greengenes database (Version 13.8, available at http://greengenes.secondgenome.com) was employed and appropriate alignments were carried out. Principal coordinate analysis (PCoA) was executed utilizing the Bray–Curtis Dissimilarity metric to analyze the data. A linear discriminant analysis effect size (LEfSe) was conducted to show the maximum difference in the microbial structures between groups (LEfSe version 1.1, https://github.com/SegataLab/Lefse). Sequence reads from the original sequence were uploaded to NCBI’s Sequence Read Archive under accession number PRJNA1090261.

### Statistical analysis

Statistical analysis was performed using SPSS 19.0 statistical software. The significant differences among groups were analyzed by one-way ANOVA, and the Duncan’s multiple range test was used when the difference between groups was significant (*p* < 0.05). In addition, 0.05 < *p* < 0.1 were considered a trend. The results are reported as means ± standard deviations. Linear and quadratic terms of AOE on various indicators of pigs were also evaluated.

## Results

### Effect of dietary AEO level on growth performance in pigs

The effects of different dietary contents of AEO on the growth performance of finishing pigs are shown in Table 3. There were no significant differences in the average initial weights between groups. Dietary inclusion of different doses of AEO in finishing pigs had minimal impact on the average final weight, ADG, and ADFI (*p* > 0.05), compared to the control group. However, the F/G of the 1,000 mg/kg AEO treatment groups decreased by 4% compared to the CTC treatment numerically, although the positive effects did not reach statistical significance.

**Table 3:** Effect of dietary AEO levels on growth performance in pigs.

Items	CTC level, mg/kg	AEO levels, mg/kg				*p*-value	*p*-value	
	75	0	250	500	1,000		Linear	Quadratic
Average initial weight /(kg)	82.03 ± 2.46	82.69 ± 2.84	80.22 ± 3.58	82.17 ± 4.75	81.83 ± 2.98	0.773	0.772	0.397
Average final weight /(kg)	107.63 ± 2.91	107.36 ± 3.32	107.47 ± 2.57	107.97 ± 4.27	108.39 ± 3.11	0.983	0.591	0.823
ADG /(kg)	0.74 ± 0.06	0.72 ± 0.06	0.79 ± 0.06	0.75 ± 0.08	0.77 ± 0.09	0.464	0.269	0.368
ADFI /(kg)	2.62 ± 0.09	2.50 ± 0.16	2.69 ± 0.11	2.65 ± 0.15	2.62 ± 0.19	0.246	0.160	0.114
F/G	3.55 ± 0.25	3.50 ± 0.09	3.41 ± 0.14	3.56 ± 0.22	3.41 ± 0.16	0.454	0.635	0.867

In the same row, values with different small letter superscripts mean a significant difference (*p* < 0.05), the same as in the following.

### Effect of dietary AEO level on serum biochemical parameters in pigs

The effects of different AEO levels in diet on serum physiological and biochemical parameters of finishing pigs are presented in Table 4. Dietary supplementation with AEO significantly increased the serum L-DLC content compared to the CTC group (*p* < 0.05). Supplementation of AEO exhibited a tendency to increase the serum GLB content of pigs (*p* = 0.088), and there was a linear improvement in the serum total protein with increasing AEO levels (*p* < 0.05). Dietary supplement with AEO had minimal impact on serum GLU, TG, TCHO, UREA, TP, ALB, H-DLC, ALP, AST and ALT contents compared to the control treatment and CTC addition treatment (*p* > 0.05).

**Table 4:** Effect of dietary AEO levels on serum biochemical indices in pigs.

Items	CTC level, mg/kg	AEO levels, mg/kg				*p*-value	*p*-value	
	75	0	250	500	1,000		Linear	Quadratic
GLU/(mmol/L)	2.68 ± 0.67	2.46 ± 0.88	2.71 ± 0.48	2.19 ± 0.31	2.38 ± 0.30	0.701	0.540	0.901
TG/(mmol/L)	0.50 ± 0.20	0.50 ± 0.15	0.63 ± 0.11	0.59 ± 0.25	0.56 ± 0.14	0.780	0.690	0.379
TCHO/(mmol/L)	2.54 ± 0.17	2.16 ± 0.71	2.31 ± 0.69	1.92 ± 0.17	2.03 ± 0.67	0.554	0.585	0.935
UREA/(mmol/L)	4.93 ± 1.31	3.50 ± 0.97	5.11 ± 1.44	5.05 ± 0.55	4.91 ± 0.48	0.197	0.073	0.088
TP/(g/L)	65.11 ± 7.53	58.63 ± 10.37	63.51 ± 5.79	64.85 ± 7.32	72.85 ± 5.17	0.166	0.021	0.682
ALB/(g/L)	24.19 ± 1.88	22.19 ± 5.99	21.17 ± 1.09	20.98 ± 2.96	24.11 ± 2.74	0.520	0.509	0.278
GLB/(g/L)	40.93 ± 5.93	36.43 ± 6.44	42.34 ± 5.22	43.87 ± 4.63	48.74 ± 6.01	0.088	0.010	0.857
H-DLC/(mmol/L)	0.88 ± 0.07	0.84 ± 0.10	0.89 ± 0.16	0.84 ± 0.17	0.86 ± 0.11	0.970	0.940	0.725
L-DLC/(mmol/L)	1.11 ± 0.16^a^	0.93 ± 0.22^ab^	0.80 ± 0.15^b^	0.74 ± 0.13^b^	0.82 ± 0.14^b^	0.047	0.229	0.365
ALP/(U/L)	74.53 ± 5.75	79.14 ± 14.86	80.65 ± 9.67	72.98 ± 7.51	82.10 ± 8.08	0.627	0.959	0.481
AST/(U/L)	20.75 ± 3.75	18.93 ± 3.64	20.57 ± 7.47	21.02 ± 3.45	20.25 ± 2.33	0.967	0.677	0.612
ALT/(U/L)	55.65 ± 10.51	42.75 ± 11.37	44.28 ± 12.32	37.81 ± 6.57	43.96 ± 6.97	0.186	0.898	0.641

In the same row, values with different small letter superscripts mean a significant difference (*p* < 0.05), the same as in the following.

### Effect of dietary AEO levels on serum antioxidant parameters in pigs

As shown in Table 5, the supplementation of AEO at doses of 250 and 500 mg/kg AEO had a tendency to increase the serum total antioxidant capacity levels in pigs (*p* = 0.060). Furthermore, the activity of glutathione (GSH) in the serum increased linearly and quadratically (*p* < 0.05) with increasing dietary concentrations of AEO from 0 to 1,000 mg/kg. The SOD and the content of GSH-Px, CAT and MDA in serum of pigs were not significantly affected (*p* > 0.05).

**Table 5:** Effect of dietary AEO levels on serum antioxidant indices in pigs.

Items	CTC level, mg/kg	AEO levels, mg/kg				*p*-value	*p*-value	
	75	0	250	500	1,000		Linear	Quadratic
SOD/(U/ml)	35.19 ± 16.78	41.69 ± 10.11	40.01 ± 7.94	38.68 ± 8.73	40.78 ± 6.42	0.918	0.833	0.662
GPX/(U/ml)	1751.23 ± 343.41	1553.69 ± 239.55	1708.31 ± 220.69	1781.56 ± 222.59	1750.18 ± 272.26	0.755	0.204	0.613
CAT/(U/ml)	4.19 ± 1.07	4.65 ± 1.35	5.23 ± 0.83	5.65 ± 0.63	5.13 ± 0.55	0.265	0.296	0.361
GSH/(μmol/L)	43.37 ± 11.71	37.70 ± 9.95	49.02 ± 4.61	52.60 ± 4.98	44.90 ± 3.90	0.123	0.045	0.026
T-AOC/(mmol/L)	1.17 ± 0.13	1.31 ± 0.05	1.35 ± 0.07	1.32 ± 0.08	1.28 ± 0.05	0.060	0.662	0.180
MDA/(nmol/ml)	2.55 ± 0.86	2.58 ± 0.58	2.30 ± 0.62	2.35 ± 0.65	2.33 ± 0.68	0.958	0.576	0.712

In the same row, values with different small letter superscripts mean a significant difference (*p* < 0.05), the same as in the following.

### Effect of dietary AEO levels on serum immune index and in pigs

The effects of AEO on serum complement C3 and C4 parameters and immunoglobulins in finishing pigs are presented in Table 6. Dietary supplementation with AEO and CTC had a tendency to increase the serum IgG levels (*p* = 0.083), and the IgG concentration in serum increased linearly as the dietary AEO concentrations increased from 0 to 1,000 mg/kg (*p* < 0.05). Additionally, the IgA, IgM, C3 and C4 concentration showed no significant differences among all treatments (*p* > 0.05).

**Table 6:** Effect of dietary AEO levels on serum immune indices in pigs.

Items	CTC level, mg/kg	AEO levels, mg/kg				*p*-value	*p*-value	
	75	0	250	500	1,000		Linear	Quadratic
IgA (μg/mL)	641.90 ± 36.78	634.34 ± 17.11	639.10 ± 25.03	642.82 ± 26.42	638.49 ± 55.82	0.997	0.811	0.844
IgG (mg/mL)	19.60 ± 1.73	17.24 ± 1.63	19.14 ± 0.92	19.22 ± 0.92	19.90 ± 1.07	0.083	0.007	0.555
IgM (mg/mL)	19.70 ± 0.72	19.93 ± 1.39	19.54 ± 1.55	19.01 ± 1.35	19.02 ± 1.82	0.846	0.356	0.963
C3 (μg/mL)	107.26 ± 12.24	103.37 ± 7.09	103.29 ± 1.89	100.52 ± 6.97	102.56 ± 2.90	0.764	0.658	0.850
C4 (μg/mL)	7.62 ± 0.85	7.62 ± 0.70	7.35 ± 0.46	7.41 ± 0.78	7.53 ± 1.59	0.991	0.869	0.709

In the same row, values with different small letter superscripts mean a significant difference (*p* < 0.05), the same as in the following.

### Effect of dietary AEO levels on nutrient apparent digestibility in pigs

Both AEO and CTC tended to increase dry matter digestibility compared to the control group (*p* = 0.086, Table 7). The dose of AEO showed a quadratic relationship with the apparent digestibility of crude ash and calcium (*p* < 0.05). Additionally, CTC tend to improve the apparent digestibility of crude fiber (*p* = 0.065), and significantly increased the apparent digestible energy of the feed (*p* < 0.05). Neither AEO nor CTC had an effect on the digestibility of CP, EE, GE and phosphorus (*p* > 0.05).

**Table 7:** Effect of dietary AEO levels on nutrient apparent digestibility in pigs.

Items	CTC level, mg/kg	AEO levels, mg/kg				*p*-value	*p*-value	
	75	0	250	500	1,000		Linear	Quadratic
DM	84.09 ± 0.57	82.18 ± 1.14	83.53 ± 0.96	83.37 ± 1.82	83.33 ± 0.78	0.086	0.108	0.324
CP	82.03 ± 1.08	80.49 ± 1.22	81.88 ± 1.65	80.31 ± 4.59	81.08 ± 1.95	0.663	0.816	0.679
EE	87.29 ± 1.02	87.84 ± 1.34	86.94 ± 1.49	86.85 ± 1.93	87.86 ± 0.26	0.662	0.688	0.201
Ash	34.95 ± 2.05ab	30.51 ± 1.88c	34.62 ± 4.52abc	37.08 ± 1.27a	31.65 ± 4.04bc	0.021	0.157	0.020
*CF*	44.95 ± 5.51	38.20 ± 6.48	37.69 ± 2.21	36.15 ± 2.98	37.69 ± 2.72	0.065	0.690	0.745
Ca	39.99 ± 2.76^a^	34.33 ± 3.47^b^	43.45 ± 5.31^a^	41.38 ± 4.03^a^	38.99 ± 1.60^ab^	0.014	0.038	0.011
P	48.41 ± 3.81	46.89 ± 2.84	45.32 ± 5.26	45.33 ± 3.11	44.38 ± 9.17	0.730	0.472	0.967
GE	86.38 ± 0.68	85.46 ± 1.23	85.86 ± 0.71	85.62 ± 1.97	85.44 ± 0.66	0.630	0.962	0.606
AME (Mcal/kg)	3.27 ± 0.03^a^	3.17 ± 0.06^b^	3.16 ± 0.03^b^	3.17 ± 0.09^b^	3.15 ± 0.02^b^	0.006	0.734	0.851

In the same row, values with different small letter superscripts mean a significant difference (*p* < 0.05), the same as in the following.

### Alteration of dietary AEO levels on gut microbiota in pigs

**Figure 1:**
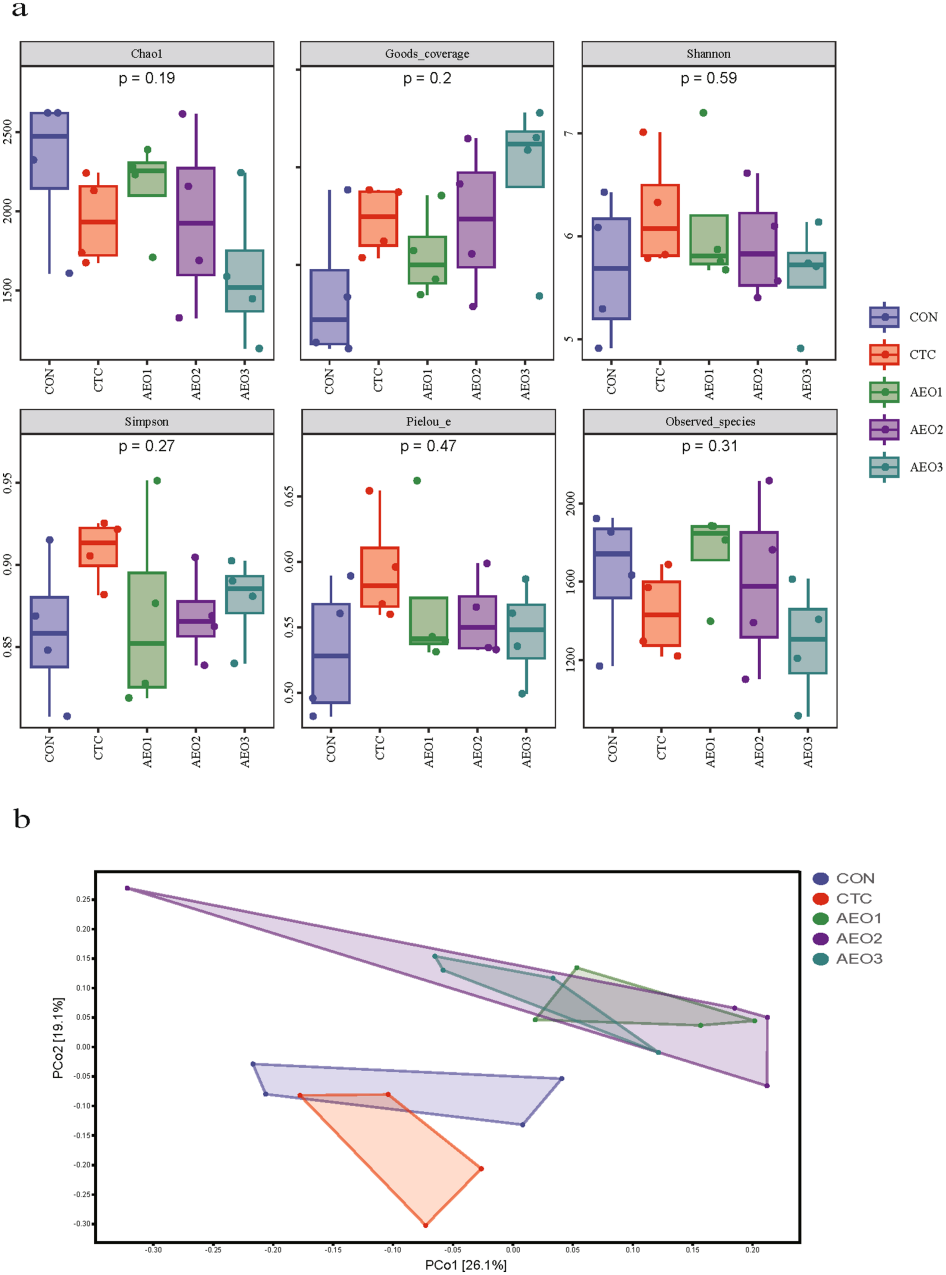
**(a)** Alpha diversity indices of the fecal bacterial communities in pigs. Con represents the group fed a basal diet, and CTC represents the group fed a basal diet with antibiotics, AEO1, AEO2, and AEO3 combined with 250 mg/kg, 500 mg/kg, and 1,000 mg/kg, respectively. **(b)** Principal coordinates analysis (PCoA) of fecal contents bacterial community of pigs. Con represents the group fed a basal diet, and CTC represents the group fed a basal diet with antibiotics, AEO1, AEO2, and AEO3 combined with 250 mg/kg, 500 mg/kg, and 1,000 mg/kg, respectively.

The rarefaction curves nearly reached the saturation plateau (Supplementary Figure S1), indicating that the sequencing captured most of the bacterial diversity. The gut microbiota community did not exhibit significant differences for the *α*-diversity index (Figure 1a), but *β*-diversity index revealed that AEO administration differed significantly from both the CON and CTC groups (Figure 1b).

**Figure 2:**
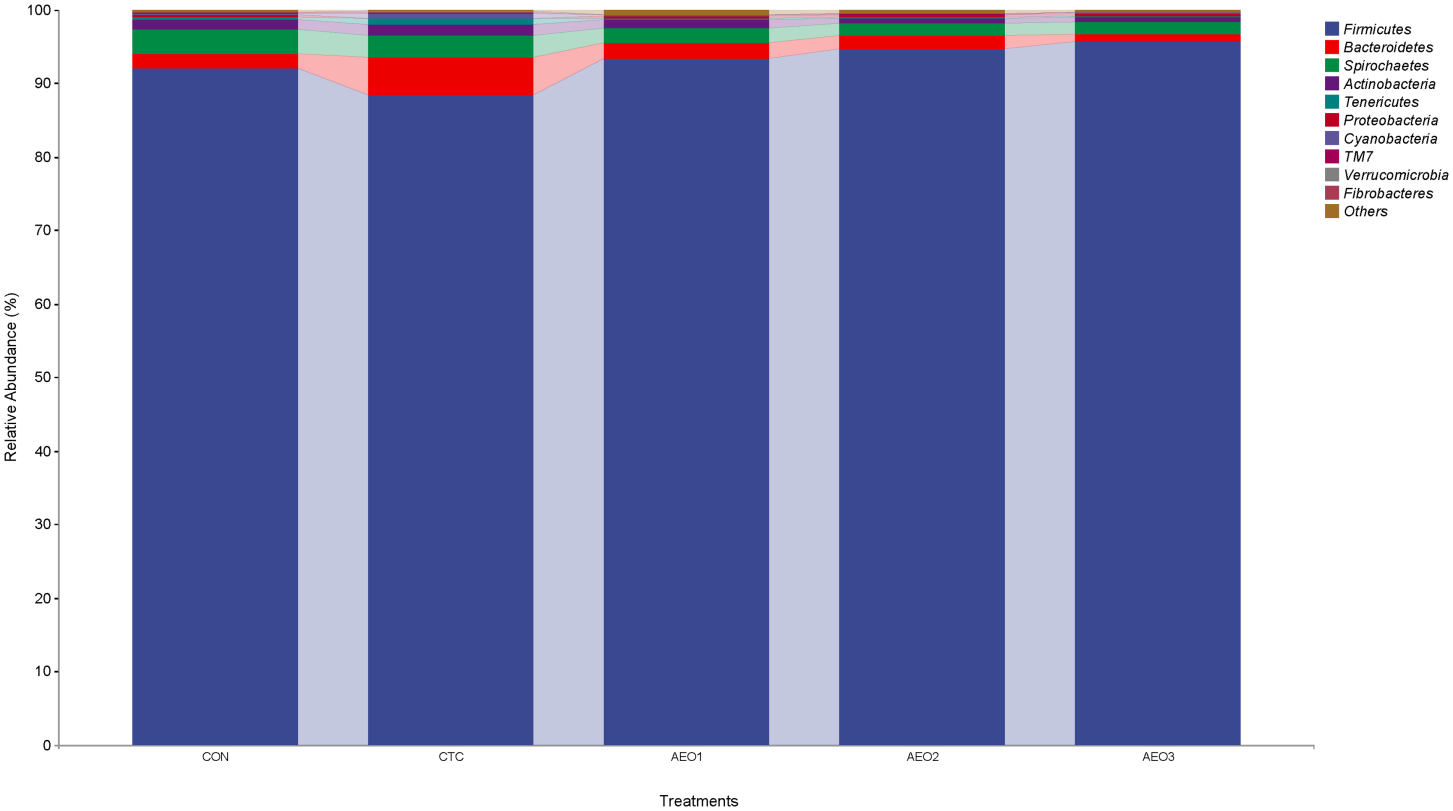
Distribution of fecal bacteria at the phylum level in pigs. Con represents the group fed a basal diet, and CTC represents the group fed a basal diet with antibiotics, AEO1, AEO2, and AEO3 combined with 250 mg/kg, 500 mg/kg, and 1,000 mg/kg, respectively.

**Figure 3:**
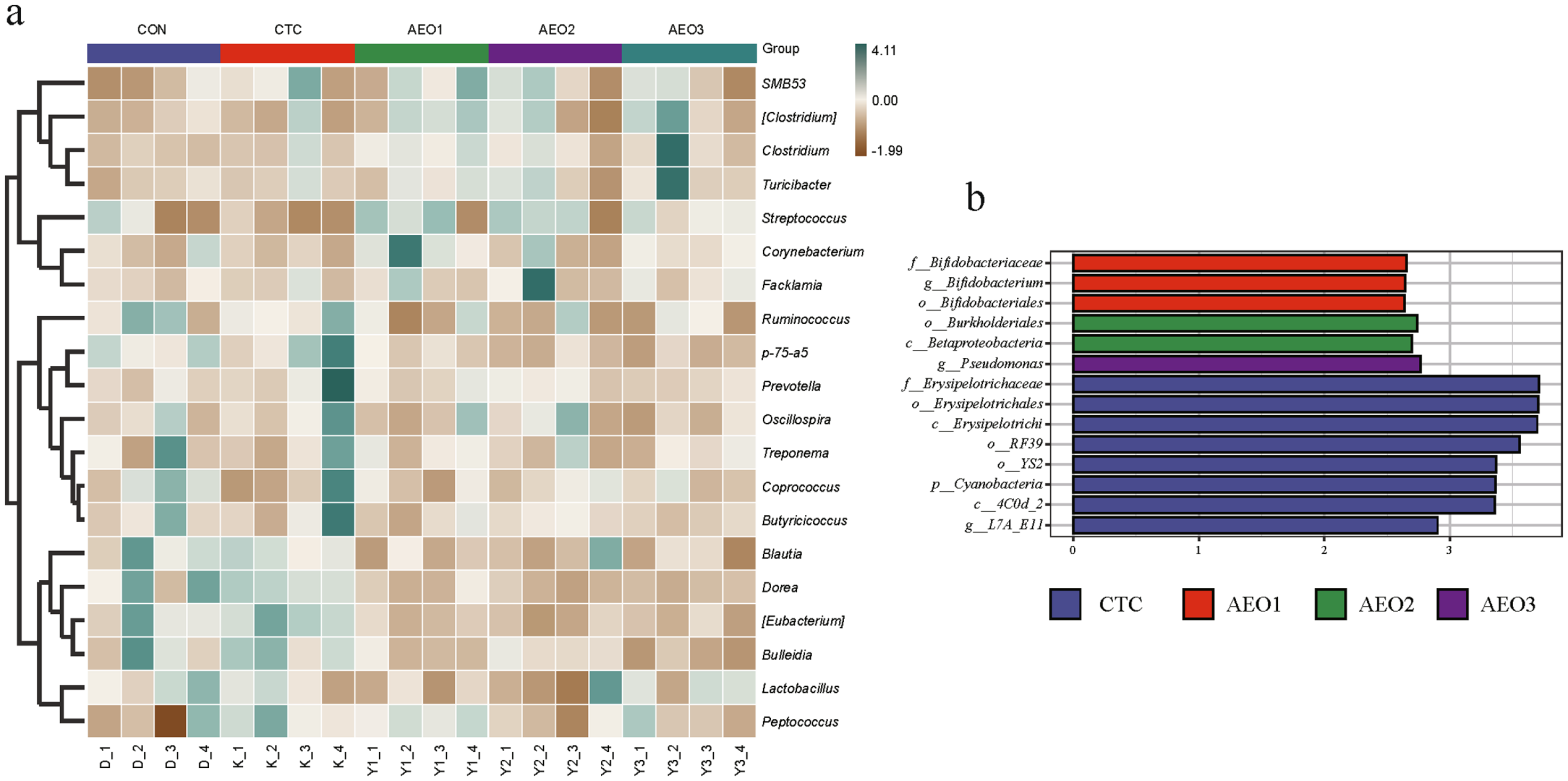
Effects of different concentrations AEO on fecal bacteria at the genus level in pigs. **(a)** Distribution of fecal top 30 bacteria at the genus level in pigs. **(b)** Differential microbe composition between the study groups determined by LEfSe. Con represents the group fed a basal diet, and CTC represents the group fed a basal diet with antibiotics, AEO1, AEO2, and AEO3 combined with 250 mg/kg, 500 mg/kg, and 1,000 mg/kg, respectively.

In the pig gut microbiota, microorganisms at the phylum level mainly consist of *Firmicutes*, *Bacteroidetes*, *Spirochaetes*, and *Actinobacteria* (Figure 2). The heatmap illustrates differences in microbial composition among the five groups at the genus level (Figure 3a). The AEO groups exhibited higher relative abundances of *Dorea*, *Blautia*, *Butyricicoccus*, *Bulleidia*, and *Lactobacillus* compared to the control and CTC groups. On the other hand, the relative abundances of *Clostridium*, *Streptococcus*, and *Turicibacter* were lower. Within the *Bifidobacteriales* group, which includes *Bifidobacteriaceae* and *Bifidobacterium*, significantly higher abundances were observed in the AEO1 group (Figure 3b). *Pseudomonas* were significantly more abundant in the AEO3 group, while *Burkholderiales* and *Betaproteobacteria* were significantly more abundant in the AEO2 group (Figure 3b).

## Discussion

Generally, various stressors impact pig health, including pathogens, oxidative stress, inflammation, and reduced growth performance (9). Dietary essential oils have been shown to support pig health through several biological mechanisms, such as anti-inflammatory, antioxidant, antimicrobial, feed palatability enhancement, gut microbiome alteration, and immunomodulatory activities (6, 25–28). The current study aims to evaluate the effectiveness of different concentrations of AEO in improving growth performance, serum antioxidant capacities, immune function, apparent digestibility of nutrients, and gut microbiota in fattening pigs. The results show that different concentrations of AEO had no statistical effects on growth performance and feed efficiency in pigs. However, other studies have corroborated the positive effects of essential oils on pig health and growth performance, as varied compounds present in essential oils contributed to the fragrance of the oil, increasing feed palatability and improving feed intake eventually (29, 30). In this study, the supplementation of 1,000 mg/kg AEO in the diet might result in better growth performance in finishing pigs.

Serum biochemical parameters usually serve as indicators of nutritional metabolism and organ functions in animals (31). The levels of TP, ALB, and GLB in serum are commonly used to assess liver function and the nutritional status of animals (32). Serum TP concentration is closely associated with nitrogen digestibility and protein synthesis capacity (33, 34). Therefore, improved nitrogen digestibility and protein synthesis capacity in animals can lead to a reduction in fecal noxious gas concentration and subsequent decrease in fecal gas emission. Furthermore, GLB, comprising the alpha, beta, and gamma globulin fractions based on protein electrophoretic fractionation, can also reflect immune response and serve as an evaluation point in routine toxicity studies (35, 36). In the present study, when supplemented with AEO in dietary, pigs had higher serum TP, relatively higher GLB and lower L-DLC. This suggests that AEO contributes to better serum biochemical parameters in pigs, and the enhanced capacity for protein and energy metabolism may result in a higher lean meat percentage and better health in lean pigs. These results are consistent with previous studies reported by Su. et al. (6, 37). Besides, when supplemented with AEO, a trend of improved serum IgG concentration has been observed in this research, suggesting enhanced immune function in pigs.

Cellular redox homeostasis is intricately maintained through the generation and elimination of reactive oxygen species (ROS). These ROS not only have the potential to induce cellular damage through the oxidation of proteins, lipids, and DNA, but also serve as signaling molecules that regulate transcription factors and epigenetic pathways, ultimately influencing cell survival and apoptosis (38). Cells in a healthy body usually maintain a redox balance between the generation of reactive oxygen species (ROS) and ROS elimination (39). Many environmental stressors, such as weaning, bacterial and viral infections, and heat, continually impose extensive antioxidant pressures on pigs. These stressors result in ROS accumulation and oxidative stress, putting animals under metabolic stress (40). Under metabolic stress, the redox balance becomes vulnerable and easily damaged (41). Therefore, these findings highlight the importance of antioxidant capacity. In this study, when supplemented with AEO, the pigs exhibited higher enzyme activity in T-AOC and GSH. Total antioxidant capacity is a primary measurement to evaluate the state and potential of oxidative stress, and GSH has been reported to efficiently maintains cellular redox balance (42, 43). These antioxidant capacities, which are involved in the NF-E2-related factor (Nrf2) pathway, could also have hepatic protective effects under pathological conditions (44). Furthermore, meat quality is closely related to the body’s antioxidant status (45), which is also a major factor affecting the consumers’ assessment. Hence, dietary supplementation with AEO has the potential to enhance the body’s antioxidant capacity, effectively protecting pigs from oxidative stress and alleviate metabolic stress.

Digestive enzymes in the intestinal tract play a crucial role in the digestion and absorption of nutrients. Previous studies have demonstrated that essential oils can improve the activity of digestive enzymes and improved nutrient digestibility eventually, which aligns with the findings of this study (46). For instance, Peng et al. (47) reported that dietary supplement with essential oil increased the ratio of villus height to crypt depth in duodenum and sucrase activity in the jejunum mucosa. Furthermore, essential oils were found to up-regulate claudin-1 and IGF-2 mRNA levels and down-regulate TRAF-6, TNFSF15, and TOLLIP mRNA levels in the jejunum of broilers, exhibiting anti-apoptotic and anti-inflammatory effects (48). Essential oils consist of phenolics and aromatic compounds, which enhance feed palatability and intake due to their better flavor and odor (49). these compounds stimulate digestive enzyme secretion and oronasal sensing, specifically for appetite regulation (50–52). Moreover, essential oils have been shown to improve intestinal morphology in pigs, potentially leading to enhanced nutritional absorption and digestion (53). In our study, both CTC and 250 mg/kg of essential oils improved nutrient digestibility, including dry matter, crude ash, calcium, and digestible energy.

The diversity and stability of the gut microbiota are intimately linked to a balanced and stable microecological barrier. A healthy digestive system contributes to improved growth performance (45). The PCoA plots of beta diversity demonstrated that both dietary CTC and AEO altered the composition of the gut microbiota, with AEO showing a greater significant impact. At the phylum level, treatment with CTC and AEO led to a decrease in the relative abundance of *Proteobacteria*, which includes various pathogens and opportunistic pathogens such as *Escherichia*, *Salmonella* and *Vibrio* (54). Research has also shown a positive correlation between *Proteobacteria* and gut inflammation in different mouse models of colitis (55, 56). The reduced abundance of *Proteobacteria* may decrease susceptibility to inflammatory conditions in the intestinal barrier of animals. Additionally, the gut microbiota of pigs are dominated by *Firmicutes* and *Bacteroidetes*, similar to humans (57). CTC also reduced the *Firmicutes* to *Bacteroidetes* ratio, a relevant marker of gut dysbiosis and obesity (58, 59).

A balanced gut microbiota forms a natural barrier on the surface of the intestinal mucosa and participates in normal digestion and absorption in animals. It also regulates immune function and prevents the invasion of pathogenic bacteria and opportunistic pathogen (60, 61). The *Blautia* is a genus of anaerobic bacteria exhibiting probiotic characteristics, widely found in the feces and intestines of mammals (62). It primarily provides beneficial anti-inflammatory effects (63). Pigs that were fed with AEO also had a high abundance of *Lactobacillus* in gut microbiota, which has a long history as an exogenous probiotic (64). Similarly, *Butyricicoccus*, a kind of butyrate producer with probiotic potential and abundant in AEO group, has been reported to have a positive impact on gut health. These gut microbes play a crucial role in digesting dietary crude fiber and producing short-chain fatty acids (SCFAs), which offer various health benefits to the host (65, 66). SCFAs can bind and activate G protein-coupled receptors (GPRs) such as GPR41 and GPR43, which are expressed in the gastrointestinal tract (67). These receptors have several health benefits, including regulating glucose metabolism, improving insulin sensitivity, and reducing inflammation (68). Specifically, butyrate, a type of SCFA, can initiate a epigenetic program in macrophages, resulting in a reduction in glycolysis and mTOR signaling while promoting the maturation of autophagosome-lysosome (69). In summary, the metabolite products of probiotics have a positive impact on maintaining gut homeostasis. Furthermore, the LEfSe analysis also revealed that some probiotics were abundant in AEO groups. The *Bifidobacterium*, for instance, positively influences gut health by aiding digestion, producing essential compounds such as B vitamins and healthy fatty acids, and occupying a niche to prevent infections by pathogens (70, 71). The *Pseudomonas* is known to play a role in breaking down complex carbohydrates and producing short-chain fatty acids in pigs, contributing to gut health maintenance (72). However, research suggests that some microbes in AEO groups may have both beneficial and potentially harmful effects on health, which are not yet well understood, including *Dorea*, *Bulleidia*, *Burkholderiales* and *Betaproteobacteria*. *Dorea* may prevent food allergies and sensitivities in infants (73), and prevent obesity and insulin resistance (74). A recent study has also shown that high bacterial abundances of *Dorea* in the gut microbiome are linked to expansion, immune checkpoint expression, and efficacy of CD19-directed CAR T-cells in patients with relapsed/refractory Diffuse-Large B-Cell Lymphoma (75). Among the decreased relative abundance in the gut microbiota of pigs influenced by AEO, *Streptococcus* is a commonly found bacterial pathogen in pigs (76). For *Clostridium*, research in pigs is limited, and its impact varies depending on the specific species. For example, a research reported that *Clostridium perfringens PLC* could the trigger ERK1/2 pathway to cause cytotoxicity (77). Conversely, the enrichment of Clostridium subgroups may contribute to the improvement of T1D and associated immune imbalance (78). In addition, it has been found that *Turicibacter* is considered a pro-inflammatory bacteria, and its abundance increases during an enteritis episode (79). Overall, these results demonstrate that AEO modulated the gut microbiota of fattening pigs by increasing the abundance of probiotics and decreasing the abundance of opportunistic pathogens.

## Conclusion

Dietary supplementation of 1,000 mg/kg AEO in the pigs’ diet has the potential to improve growth performance and various physiological and biochemical indicators. AEO can also enhance antioxidant levels and increase the efficiency of nutrient digestion and absorption through the regulation of gut microbiota. These findings suggest that AEO may be a viable alternative to CTC.

## Data Availability

The datasets presented in this study can be found in online repositories. The names of the repository/repositories and accession number(s) can be found at: https://www.ncbi.nlm.nih.gov/, PRJNA1090261.

## Supplementary material

Supplementary Figure S1 Rarefaction curves of observed bacterial sequences in the fecal contents of pigs. Con represents the group fed a basal diet, and CTC represents the group fed a basal diet with antibiotics, AEO1, AEO2, and AEO3 combined with 250 mg/kg, 500 mg/kg, and 1000 mg/kg, respectively.
