# Supplementary figures and images for: The effect of Alpinia oxyphylla essential oil on growth performance, immune, antioxidant functions and gut microbiota in pigs
Source: Front Vet Sci. 2024 Dec 10;11:1468520. doi: 10.3389/fvets.2024.1468520 (PMC11666522; doi:10.3389/fvets.2024.1468520)

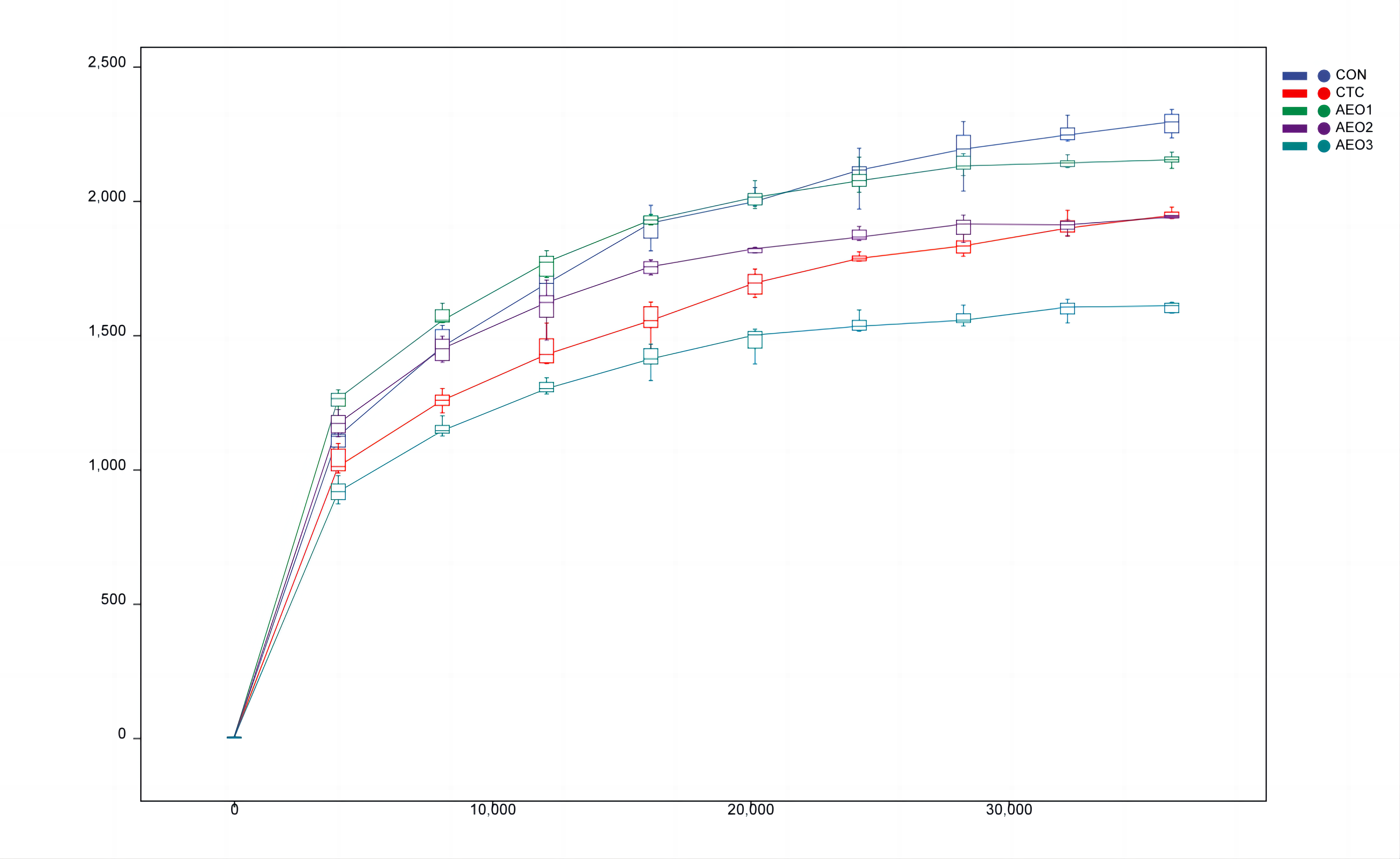

Supplement: Supplementary Figure S1 — Rarefaction curves of observed bacterial sequences in the fecal contents of pigs. Con represents the group fed a basal diet, and CTC represents the group fed a basal diet with antibiotics, AEO1, AEO2, and AEO3 combined with 250 mg/kg, 500 mg/kg, and 1000 mg/kg, respectively. [file Image_1.TIF]
